# Decreased Serum 25-hydroxyvitamin D Level Causes Interventricular Septal Hypertrophy in Patients on Peritoneal Dialysis: Cardiovascular Aspects of Endogenous Vitamin D Deficiency

**DOI:** 10.1155/2016/2464953

**Published:** 2016-11-27

**Authors:** Bennur Esen, Irfan Sahin, Ahmet Engin Atay, Emel Saglam Gokmen, Ozlem Harmankaya Kaptanogullari, Mürvet Yılmaz, Suat Hayri Kucuk, Serdar Kahvecioglu, Nurhan Seyahi

**Affiliations:** ^1^Department of Internal Medicine and Nephrology, Bagcilar Education and Research Hospital, Istanbul, Turkey; ^2^Department of Cardiology, Bagcilar Education and Research Hospital, Istanbul, Turkey; ^3^Department of Internal Medicine, Bagcilar Education and Research Hospital, Istanbul, Turkey; ^4^Department of Internal Medicine and Nephrology, Bakırkoy Sadi Konuk Education and Research Hospital, Istanbul, Turkey; ^5^Department of Biochemistry, Bagcilar Education and Research Hospital, Istanbul, Turkey; ^6^Department of Internal Medicine and Nephrology, Sevket Yılmaz Education and Research Hospital, Bursa, Turkey; ^7^Department of Internal Medicine and Nephrology, Cerrahpasa School of Medicine, Istanbul University, Istanbul, Turkey

## Abstract

*Introduction*. In the present study, we aimed to analyze the relation of vitamin D with echocardiographic indexes in patients with end stage renal disease (ESRD) receiving renal replacement therapy (RRT).* Methods*. A total of 98 patients, 64 patients on hemodialysis (HD) (29F/35M, mean age 56.75 ± 18.63 years) and 34 age matched patients on peritoneal dialysis (PD) (21F/13M, mean age 58.11 ± 10.63 years), with similar duration of ESRD and RRT were enrolled into this cross-sectional study. Echocardiographic examination was performed after dialysis session at normovolemic status. Fasting blood samples were obtained before dialysis session.* Results*. Patients on PD and female patients in both groups had significantly lower level of 25-OH-D3 level when compared to patients on HD or male patients (*p*: 0.0001 and* p*: 0.0001). When all participants were considered, there was no significant association between 25-OH-D3 and echocardiographic parameters; however, in patients on PD, a significant negative correlation was determined between 25-OH-D3 and diastolic blood pressure, interventricular septal hypertrophy (ISH), and left ventricular mass index (LVMI) (*r*: −0.424,* p*: 0.012;* r*: −0.508,* p*: 0.004;* r*: 0.489,* p*: 0.04, resp.).* Conclusion*. Low serum 25-hydroxyvitamin D levels is associated with ISH and LVMI in PD patients.

## 1. Introduction

Besides its action on bone-mineral metabolism, vitamin D has numerous physiologic effects including cell growth and differentiation of immune system [1]. Also vitamin D deficiency (VDD) has a well-known association with endothelial dysfunction [2]. In vitro studies indicated a favourable antiproliferative effect of vitamin D replacement on relaxation of cardiomyocytes and diastolic functions of heart [3, 4].

Cardiovascular disorders (CVD) account most common cause of mortality in ESRD. On the other hand, chronic kidney disease (CKD) is associated with accelerated development of CVD [5]. Patients with CKD tend to have accelerated atherosclerosis, valvular calcification, asymmetric septal hypertrophy, and arrhythmias when compared to general population. Innovations in renal replacement procedures prolong survival in ESRD patients and consequent increase in CVD risk [6].

Vitamin D receptors have been identified in several extrarenal organ systems including cardiac myocytes and endothelial cells [7]. In animal studies, researchers have demonstrated an association between VDD and increased contractility and deteriorated systolic functions, both leading to myocardial hypertrophy [8]. Mose et al. showed that active vitamin D therapy for 6 months improved end-diastolic volume of left ventricule [9]. Similarly, active vitamin D and vitamin D receptor activators (paricalcitol) are related to decreased mortality and better cardiovascular risk profile. Low serum vitamin D level cause hypocalcemia and secondary hyperparathyroidism that are related to coronary artery calcification, cardiac failure, and mortality [8].

There is a strict relationship between VDD and CVD-related mortality; however exact mechanism is not clearly understood. We aimed to examine the effect of 25-OH-D3 on echocardiographic indices and cardiac functions in ESRD patients receiving RRT.

## 2. Material-Method

A cross-sectional study on 98 ESRD patients receiving RRT was conducted between January 2015 and March 2015 in Bagcilar Education and Research Hospital. Patients were divided into 2 subgroups: 64 patients on hemodialysis (HD) and 34 age matched patients on peritoneal dialysis (PD) with similar duration of ESRD and RRT. Ethics committee of Bagcilar Education and Research Hospital approved the study. Written informed consent was obtained from all participants.

Entire patients in each group were under dietary restrictions that is recommended by K/DOQI for patients on dialysis therapy [10]. Patients on PD and HD were regularly followed up in Dialysis Center and Nephrology outpatient service of Bagcilar Education and Research Hospital. Serum potassium, sodium, calcium, and hemogram analysis were performed by monthly intervals, and parathormon level was analyzed by 3-month intervals. Patients were informed about the results and, consequently, recommendations were updated by special dietitian. Also their first-degree relatives (parents, husbands, wives, or children) were integrated to follow-up procedure to augment patient's continuity and accommodation. Similarly, patients in both groups were advised with exposure to sunlight not less than 1 hour/day. The study was performed between January and March 2015 in Istanbul to eliminate variations of seasonal exposure to sunlight. All the patients have the same ethnic background and were residents of the same region in European side of Istanbul city. All the patients survived during the study period

Exclusion criteria were anemia, obesity, acute inflammatory or infectious disease, malignancy, immobility, ongoing treatment with immunosuppressive agents, severe hypertension, hypohyperparathyroidism, pregnancy, left ventricular ejection fraction (LVEF) <50%, moderate-to-severe valvular disease, or age younger than 18 years. Data regarding demographic features and physical examination were recorded.

Blood samples were collected after 8-hour fasting period. Serum levels of calcium (Ca), phosphorus (P), albumin, alkalen phosphatase (ALP), and low density lipoprotein (LDL) were analyzed by photometric method in Siemens Advia 1800 device. Electrochemiluminescence immunoassay (ECLIA) method was used to analyze serum levels of parathormon (PTH) and 25-hydroxyvitamin D3. Vitamin D deficiencies were classified as severe, <5 ng/mL; moderate, 5–15 ng/mL; and mild, 15–29 ng/mL [11]. Vitamin D replacement was instituted on the basis of achieving a PTH level of <300 IU/L as recommended by K/DOQI [10]. The use of vitamin D replacement was recorded for each patient.

Echocardiographic examination was performed after dialysis session at normovolemic status. Normovolemia or dry weight was defined as postdialysis weight where the patient shows no sign of pulmonary or peripheral edema and do not have hypotension [12]. Fasting blood samples were obtained before dialysis session. All cases were examined by iE33 Echocardiography system to detect left ventricular end-diastolic dimension (LVEDD), left atrial diameter (LAD), left ventricular posterior wall thickness in diastole (LVPWT), interventricular septum thickness in diastole (IVST), and left ventricular ejection fraction (LVEF). Left ventricular mass was calculated according to the American Society of Echocardiography formula. Body mass index (BMI) was calculated by dividing weight into the square of height, body surface area (BSA) was calculated as ([height (cm) × weight (kg)]/3600)^1/2^, and LV mass index was calculated by dividing LV mass into the BSA. Left ventricular hypertrophy was defined as LV mass index >115 g/m^2^ (for men) and >95 g/m^2^ (for women) [13]. The use of angiotensin converting enzyme (ACE) inhibitors and angiotensin receptor blockers (ARB) was recorded for each patient.

### 2.1. Statistical Analysis

SPSS 22.0 (SPSS, Chicago, Illinois, USA) package programme was used for statistical analysis. Parametric variables were compared with independent* t*-test, ordinal data were compared with Mann–Whitney* U* test, and nonparametric variables were compared with chi-square test. Quantitative parameters were compared with Kruskal Wallis test. Spearman's rho correlation test was used to evaluate the relation of parametric variables. A *p* value <0.05 was considered significant.

## 3. Results

Group 1 consists of 64 patients on HD with a mean age of 56.42 ± 18.37 years and mean RRT duration of 38.12 ± 15.62 months. In group 2, there were 34 patients on PD with a mean age of 53.11 ± 10.63 years and mean RRT duration of 34.64 ± 14.59 months. The mean age and RRT duration of both groups were similar. Creatinine levels were similar in both groups. In most of the patients, daily urine output was below 200 mL/day; therefore renal clearance was negligible.

Serum levels of Ca, P, PTH, and alkalen phosphatase (ALP) were similar between patients on HD and PD. Lipid profile including LDL-c, HDL-c, and triglyceride level were similar between patients on HD and PD. Serum 25-OH-D3 level of patients on PD (4.68 ± 2.93 ng/mL) was significantly lower than patients on HD (9.29 ± 7.47 ng/mL) (*p*: 0.0001). Women in both groups had low 25-OH-D3 level when compared to male participants (*p*: 0.0001).  Table 1 shows the biochemical and demographic data of both groups.

Two patients in PD group and 3 patients in HD group were receiving statin therapy. The mean ± SD systolic blood pressure of patients on HD and PD was 118 ± 21 mmHg and 122 ± 17 mmHg, respectively. The mean ± SD diastolic blood pressure of patients on HD and PD was 70 ± 15 mmHg and 72 ± 14 mmHg, respectively. Both mean systolic and diastolic pressure of patients on HD and PD were statistically similar. The ratio of antihypertensive drug use in PD and HD group was similar. Frequency of ACE-inhibitor or ARB use was 8.2% and 6.9% for HD and PD patients respectively. Also vitamin D was administered according to parathormon level of patients as recommended by K/DOQI clinical practice guidelines for bone metabolism and disease in CKD. Frequency of active vitamin D use was 91.8% and 93.1% for HD and PD patients, respectively.

Echocardiographic parameters were similar in HD and PD group (Table 2). When all patients were considered, correlation analysis indicated a significant association between 25-OHD3 and age (*r*: −0.226,* p*: 0.028). However, there was no significant association between 25-OHD3 and echocardiographic parameters.

In HD group, there was no significant association between 25-OH-D3 and echocardiographic parameters (Table 3). In PD group, a significant inverse correlation was determined between 25-OH-D3 and diastolic blood pressure, aortic velocity (Aovel), pulmonary velocity (Pvel), aortic regurgitation (AR), interventricular septal hypertrophy (IVSH), and left ventricular mass index (*r*: −0.424,* p*: 0.012;* r*: −0.433,* p*: 0.024;* r*: −0.498,* p*: 0.006;* r*: −0.430,* p*: 0.022;* r*: −0.508,* p*: 0.004;* r*: 0.489,* p*: 0.04; resp.) (Table 4).

## 4. Discussion

Our study indicates that female participants and patients on PD have lower 25-OH-D3 level. Additionally, PD patients but not HD patients with low 25-OH-D3 level have structural cardiovascular changes which may be related to high diastolic blood pressure, ISH, and LVMI.

CVD-related mortality risk of patients with CKD varies between 40% and 50% [14]. Low serum 25-OH-D3 levels are frequently seen in CKD patients. In a study by Taskapan et al., mild, moderate, and severe VDD was observed in 43,9%, 48,4%, and 4,4%, respectively, similar to our study [15]. A growing body of evidence indicates the relation of VDD with morbidity and mortality in patients with CKD. Ravani et al. showed that 25-OH-D3 independently and more accurately predicts progression of CKD and mortality in 168 patients with stages 2–5 CKD when compared to 1,25-OH-D3 [16]. Wang and Wells demonstrated a high CV event risk in a study on 230 PD patients with low serum 25-OH-D3 level [17]. Pekkanen et al. determined a relation between decreased LVEF and low serum 25-OH-D3 level [18].

Drechsler et al. showed a 3 times increased sudden cardiac death among patients with severe VDD (<25 nmol/L) when compared to patients with adequate vitamin D status (>75 nmol/L) [19]. In “Framingham Offspring Study,” patients with low serum 25-OH-D3 and without a history of CVD have high mortality rates [20]. All cause and CV related mortality rates were significantly higher in patients with low 25-OH-D level than normal 25-OH-D in patients that underwent coronary angiography and were followed up for 7.7 years [21].

Vitamin D metabolites have renoprotective effect by antiproteinuric, anti-inflammatory, and immunomodulatory properties and by suppression of renin-angiotensin-aldosterone system (RAAS) [22]. Low serum 25-OH-D level activates (RAAS) and increases fibroblast growth factor which are associated with progression of renal injury [23, 24]. Decreased serum 25-hydroxyvitamin D level leads to IVSH which is a significant predictor of cardiovascular morbidity in ESRD in pediatric population [25]. Additionally, low serum 25-OH-D3 have been related to increased inflammatory state which is involved in atherosclerosis and CVD [26, 27].

Hypertension, DM, dyslipidemia, age, volume-nutritional status, and dialysis adequacy are well-known CVD risk factors that are frequently seen in CKD patients [27]. All these factors are correlated with echocardiographic indexes including left ventricular diastolic diameter (LVdD), LVED, and ejection fraction (EF) [27]. Because calcium-phosphorus equilibrium as well as fluid and glycemic control has significant impact on cardiac myocytes (trophic effect of PTH on cardiac myocytes), echocardiographic examinations were performed after dialysis session at normovolemic status. In the Framingham Offspring Study, individuals with low 25-OH-D3 level had a hazard ratio of 1.62 for CVD risk [27]. Because fluid control is better in HD patients, PD patients have increased CVD and mortality risk [28].

Although it is out of our study object, vitamin D replacement has favourable impact on cardiac functions and diastolic dysfunction and is associated with regression of myocardial hypertrophy [29–31]. However authors of two large scaled human studies (PRIMO and OPERA trials) and an experimental animal study failed to demonstrate a beneficial effect of vitamin D therapy on structural and functional changes in myocardium [32–34]. It would be reasonable to state that maintaining an accurate vitamin D level has vital importance in ESRD because excessive vitamin D replacement may cause hypercalcemia and hyperphosphatemia where high CaxP level is also related to calciphylaxis and vascular or valvular calcification.

Low sample size and single point measurement of variables were two major limitations of the study. A large number of dialysis patients that have exclusion criteria mentioned in the material-method section were excluded. Additionally patients with low dialysis adequacy were also excluded to eliminate the effect of hypervolemia and electrolyte imbalance on echocardiographic indices. Cross-sectional design of the study was another major limitation that should be confirmed by RCTs. To the best of our knowledge, this was the first report that indicates the association of low endogenous vitamin D status with diastolic dysfunction and ventricular hypertrophy in PD patients. In contrast to majority of previous studies, our study participants were similar in terms of CVD risk factors including blood pressure, dyslipidemia, serum glucose, age, obesity, uric acid level, and dialysis adequacy that provided us with elimination of effect of these factors on echocardiographic indices. Possible explanation of correlation between vitamin D and echocardiographic indices in PD is that fluid control and volume status were better in HD patients that have significant impact on both cardiovascular health and echocardiographic indices. Also intravenous or oral administration of vitamin D may affect serum level and echocardiographic indices. Owing to the fact that CKD patients on RRT have predisposition to CVD, 25-OH-D3 may become a candidate marker to predict future CV events. Regular follow-up of serum 25-OH-D3 may provide complementary data on cardiovascular health status of dialysis patients. Large scaled studies would clarify the exact role of vitamin D on cardiovascular morbidity and mortality in CKD patients.

## Figures and Tables

**Table 1:** Comparison of biochemical variables between groups.

	HD	PD	*p*
25-OH-D vitamin (ng/dL)	9.29 ± 7.47	4.68 ± 2.93	**0.0001**
Age (years)	56.42 ± 18.37	53.35 ± 10.49	NS
Mean duration of RRT (months)	38.12 ± 15.62	34.64 ± 14.59	NS
Kt.V	1.45 ± 0.17	2.35 ± 0.35	**NS**
Urea (mg/dL)	119.45 ± 40.19	116.34 ± 39.86	**NS**
Creatinine (mg/dL)	7.61 ± 2.71	7.47 ± 3.14	NS
Calcium (mg/dL)	8.79 ± 0.87	8.20 ± 1.33	NS
Phosphorus (mg/dL)	4.94 ± 1.67	5.30 ± 1.47	NS
PTH (pg/mL)	490.07 ± 644.92	541.4 ± 545.3	NS
Uric acid (mg/dL)	5.79 ± 1.48	6.11 ± 1.17	NS
AST (U/L)	13.98 ± 10.18	14.21 ± 9.86	**NS**
ALT (U/L)	13.79 ± 16.11	14.12 ± 9.23	**NS**
ALP (mg/dL)	119.34 ± 49.72	103.03 ± 46.86	NS
LDH (U/L)	197.14 ± 48.75	202.46 ± 53.21	**NS**
Triglyceride (mg/dL)	169.86 ± 73.57	178.45 ± 79.28	NS
Total cholesterol (mg/dL)	189.43 ± 48.52	191.36 ± 45.97	**NS**
HDL (mg/dL)	39.36 ± 14.11	41.16 ± 13.85	**NS**
LDL (mg/dL)	105.67 ± 37.68	113.23 ± 37.53	**NS**
Na (mmol/L)	137.46 ± 3.96	140.06 ± 4.7	**NS**
K (mmol/L)	4.76 ± 0.69	4.59 ± 0.71	**NS**
BMI (kg/m^2^)	26 ± 4	24 ± 5	**NS**
Systolic BP (mmHg)	117 ± 21	122 ± 17	**NS**
Diastolic BP (mmHg)	70 ± 15	72 ± 14	**NS**

**Table 2:** Echocardiographic indices of both groups.

	HD	PD	*p*
LVD, mm	47.4 ± 6.5	47.6 ± 7.4	0.878
LVS, mm	31.3 ± 6	30.2 ± 8.2	0.479
IVS, mm	12.7 ± 2.5	12.2 ± 2.4	0.310
PW, mm	12.3 ± 2.5	15.8 ± 2.36	0.212
EF, %	61.24 ± 7.09	63.77 ± 9.02	0.146
FS, %	33.3 ± 5.02	34.72 ± 5.91	0.235
EDV, ml	106.27 ± 31.99	111.27 ± 42.88	0.542
ESV, ml	41.64 ± 16.52	42.93 ± 34.5	0.815
Ao, mm	26.8 ± 2.7	26.1 ± 2.4	0.215
As.Aort, mm	32.7 ± 4.2	33.6 ± 5	0.360
LA, mm	37.2 ± 7.8	35.2 ± 6.9	0.231
Em, cm/s	59 ± 16	66 ± 24	0.175
Am, cm/s	71 ± 15	81 ± 21	**0.021**
E′, cm/s	98 ± 12	79 ± 31	0.606
Aovel, m/s	1.32 ± 0.42	1.4 ± 0.39	0.452
Pvel, m/s	0.86 ± 0.16	0.9 ± 0.16	0.291
TAPSE, cm/s	19.46 ± 4.29	18.69 ± 3.6	0.408
MR	0.8 ± 0.62	0.72 ± 0.59	0.610
AR	0.31 ± 0.5	0.41 ± 0.57	0.398
TR	1.04 ± 0.19	1.07 ± 0.25	0.506
PAPs, mmhg	27.82 ± 8.81	27.73 ± 7.89	0.964

LVD: left ventricular end-diastolic dimension, LVSD: left ventricular end-systolic dimension, IVS: interventricular septal thickness, PW: posterior wall thickness, EF: ejection fraction, FS: fractional shortening, EDV: end-diastolic volume, ESV: end-systolic volume, Ao: aortic root, As.Aort: ascendant aorta, LA: LA end-systolic dimension, Em: mitral annular early diastolic velocity, Am: mitral annular late diastolic velocity, E: tissue Doppler early diastolic mitral annular velocity, Aovel: aortic valve velocity, Pvel: pulmonary valve velocity, TAPSE: tricuspid annular plane systolic excursion, MR: mitral regurgitation, AR: aortic regurgitation, TR: tricuspid regurgitation, and PAPs: estimated pulmonary artery systolic pressure.

**Table 3:** The relation of 25-OH-D3 with echocardiographic indices in HD group.

	25-OH-D	
	*r*	*p*
LVD (mm)	−0.160	0.235
LVS (mm)	−0.089	0.516
IVS (mm)	−0.037	0.784
PW(mm)	−0.043	0.750
LV mass index	−0.210	0.230
EF (%)	−0.056	0.680
Ao (mm)	0.053	0.693
As.aort (mm)	0.165	0.248
LA (mm)	−0.082	0.546
Em (cm/s)	0.120	0.373
Am (cm/s)	0.027	0.847
E (cm/s)	−0.042	0.761
Aovel (m/s)	0.010	0.939
Pvel (m/s)	−0.063	0.649
TAPSE (cm/s)	−0.214	0.117
AR	−0.042	0.765
TPAPs (mmHg)	−0.009	0.950

LVD: left ventricular end-diastolic dimension, LVSD: left ventricular end-systolic dimension, IVS: interventricular septal thickness, PW: posterior wall thickness, EF: ejection fraction, Ao: aortic root, As.Aort: ascendant aorta, LA end-systolic dimension: Em: mitral annular early diastolic velocity, Am: mitral annular late diastolic velocity, E: tissue Doppler early diastolic mitral annular velocity, Aovel: aortic valve velocity, Pvel: pulmonary valve velocity, TAPSE: tricuspid annular plane systolic excursion, AR: aortic regurgitation, and PAPs: estimated pulmonary artery systolic pressure.

**Table 4:** The relation of echocardiographic variables with 25-OH-D3 in PD group.

	25-OH-D	
	*r*	*p*
LVD (mm)	0.081	0.669
LVS (mm)	0.103	0.590
IVS (mm)	−0.508	0.004
PW (mm)	−0.321	0.089
LV mass index (gr/m^2^)	−0.489	0.004
EF (%)	0.116	0.543
Ao (mm)	0.115	0.554
As.aort (mm)	0.195	0.301
LA (mm)	−0.119	0.539
Em (cm/s)	−0.114	0.565
Am (cm/s)	−0.213	0.258
E (cm/s)	0.352	0.061
Aovel (m/s)	−0.433	0.024
Pvel (m/s)	−0.498	0.006
TAPSE (cm/s)	−0.130	0.511
AR	−0.430	0.022
TPAPs (mmHg)	−0.221	0.250

LVD: left ventricular end-diastolic dimension, LVSD: left ventricular end-systolic dimension, IVS: interventricular septal thickness, PW: posterior wall thickness, EF: ejection fraction, Ao: aortic root, As.Aort: ascendant aorta, LA end-systolic dimension: Em: mitral annular early diastolic velocity, Am: mitral annular late diastolic velocity, E: tissue Doppler early diastolic mitral annular velocity, Aovel: aortic valve velocity, Pvel: pulmonary valve velocity, TAPSE: tricuspid annular plane systolic excursion, AR: aortic regurgitation, and PAPs: estimated pulmonary artery systolic pressure.
